# Correlation of Age and Laboratory Parameters with Urine Flow Cytometry and Culture Results in Patients with Urinary Tract Infections

**DOI:** 10.3390/idr18030056

**Published:** 2026-06-09

**Authors:** Alma Trnacevic, Emir Trnacevic, Merjema Mahmutovic, Amra Serak, Humera Porobic Jahic, Jasminka Petrovic, Dilista Piljic, Rahima Jahic, Danijel Bijedic, Amela Becirovic

**Affiliations:** 1 Clinic for Infectious Diseases, University Clinical Center Tuzla, 75 000 Tuzla, Bosnia and Herzegovina; humera.jahic@ukctuzla.ba (H.P.J.); jasminka.petrovic@ukctuzla.ba (J.P.); dilista.piljic@ukctuzla.ba (D.P.); rahima.jahic@ukctuzla.ba (R.J.); danijel_bijedic@hotmail.com (D.B.); 2 Polyclinic for Laboratory Diagnostics, University Clinical Center Tuzla, 75 000 Tuzla, Bosnia and Herzegovina; emir.trnacevic@ukctuzla.ba (E.T.); mmahmutovic91@gmail.com (M.M.); amela.becirovic@ukctuzla.ba (A.B.); 3 Public Institution Health Center Dr. Mustafa Sehovic, 75 000 Tuzla, Bosnia and Herzegovina; amraserak@yahoo.com

**Keywords:** Sysmex UF-4000, urinalysis, diagnostic stewardship, empirical antibiotic therapy, laser light scattering

## Abstract

Background: The diagnosis of urinary tract infection (UTI) remains a clinical challenge, with urine culture as the gold standard. In developing countries like Bosnia and Herzegovina, a high prevalence of antimicrobial resistance and frequent empirical treatment pose significant clinical challenges. Automated urine flow cytometry has emerged as a rapid tool to optimize diagnostic processes. Objectives: To determine the correlation of age, gender, and laboratory parameters—such as white blood cell (WBC) count, neutrophil count, and C-reactive protein (CRP)—with both urinary bacterial counts and urine culture results. Methods: This retrospective study analyzed 200 adult patients (≥18 years) with symptoms suggestive of UTI at the University Clinical Center Tuzla. Data on age, gender, WBC, neutrophils, CRP, and urine flow cytometry (Sysmex UF-4000) were collected. Statistical analysis was performed using R software (version 4.5.1), utilizing logistic regression models via the ‘glm’ function to identify independent predictors, with statistical significance set at *p* < 0.05. Results: The mean age of the population was 68.61 ± 15.19 years. Logistic regression demonstrated that WBC count (OR = 1.06, *p* = 0.004), neutrophil count (OR = 1.04, *p* = 0.014), and patient age (OR = 1.03, *p* = 0.001) were significant independent predictors of UTI. Furthermore, patients with a urinary bacterial count > 1200/μL had 83 times higher odds of a positive urine culture (OR = 83, 95% CI 32.25–200, *p* < 0.001). Conversely, CRP levels and gender were not significant predictors (*p* > 0.05). Conclusions: Patient age, WBC, and neutrophil counts are key factors for predicting UTIs. Integrating these parameters with urine flow cytometry bacterial counts can significantly enhance diagnostic accuracy and rapid screening in clinical practice.

## 1. Introduction

Urinary tract infections (UTIs) are among the most common infections worldwide, caused by the presence and multiplication of microorganisms within the urinary tract [1]. They represent a broad range of clinical and pathological states involving different segments of the urinary system. Because each clinical entity exhibits a unique epidemiological profile, distinct natural history, and specific diagnostic considerations, establishing a precise differentiation is crucial to optimize therapeutic approaches and improve patient prognosis [2]. Historically, standardized communication in this field has relied on various classification systems, most notably those established by the Centers for Disease Control and Prevention (CDC) [3], the Infectious Diseases Society of America (IDSA) [4], and the European Society of Clinical Microbiology and Infectious Diseases (ESCMID) [5].

The updated IDSA classification categorizes infections solely by clinical severity, meaning patients with diabetes or anatomical abnormalities are considered uncomplicated (uUTI) if the infection is confined to the bladder, whereas systemic symptoms or the presence of foreign devices like catheters automatically indicate a complicated infection (cUTI) [6]. In general, acute prostatitis should be considered and excluded before classifying a UTI as uncomplicated in a male patient, as prostatitis can affect the choice and duration of therapy, though prostatitis, epididymitis, and orchitis remain entirely outside the scope of these guidelines [6]. In an effort to further standardize clinical practice and offer a more robust framework for diverse clinical scenarios, the European Association of Urology (EAU) has introduced an updated classification for UTIs. This system moves away from the traditional uncomplicated versus complicated dichotomy, focusing instead on distinguishing between localized and systemic infections based on clinical presentation [2].

Although urine culture remains the diagnostic gold standard, its practical clinical utility is limited by the 24 to 48 h turnaround time for results [7]. Consequently, this delay necessitates immediate empirical antibiotic therapy, a practice that significantly drives the emergence and spread of antimicrobial resistance (AMR). In developing countries like Bosnia and Herzegovina (BiH), where infrastructure and regulatory challenges exist, AMR is highly prevalent and deep-rooted, largely due to this reliance on empirical therapy. In recent years, automated urine flow cytometry has emerged as a rapid screening tool, providing fast and reliable quantification of bacteria and white blood cells in urine (U-WBC) [8]. This technology offers a highly automated and objective approach that refines sample selection for subsequent culturing, thereby accelerating clinical decision-making. By utilizing a combination of fluorescent labeling and light scatter signals, the technique analyzes thousands of microscopic events per specimen to precisely quantify cells, microorganisms, and other debris linked to active infection [9,10].

The operational principle of this method relies on hydrodynamic focusing coupled with multi-parametric optical analysis. In this process, the uncentrifuged urine sample is automatically stained with specific polymethine fluorescent dyes that bind to cellular nucleic acids. As the stained particles pass individually through a semiconductor laser beam in a single-file stream, the instrument detects and converts three distinct optical signals into digital data: Forward Scatter (FSC) to determine particle size, Side Scatter (SSC) to evaluate internal structural complexity and granularity, and Fluorescent Light (FL) to quantify nucleic acid content. These signals generate unique optical cluster patterns (scattergrams), allowing the software to automatically differentiate and count bacteria, leukocytes, erythrocytes, epithelial cells, and casts within minutes [9,10,11]. Contemporary analyzers, such as the Sysmex UF-4000, seamlessly integrate into routine laboratory workflows, delivering these comprehensive cellular profiles in under a minute [11]. Despite its rapid processing speed, automated urinary flow cytometry exhibits notable technical limitations. Primarily, the technology lacks the capacity to differentiate viable from non-viable microorganisms, nor can it distinguish between pathogenic uropathogens and commensal/contaminant flora [11,12]. Consequently, a confirmatory quantitative urine culture remains mandatory to guide specific antimicrobial therapy.

Furthermore, the diagnostic accuracy of these systems can be influenced by factors such as patient demographics and systemic inflammatory markers. While C-reactive protein (CRP) and peripheral blood counts are commonly used to assess the severity of infections, their specific predictive value in the context of UTIs, especially when combined with flow cytometry, remains a subject of ongoing investigation.

The aim of our study was to determine the correlation between age, gender, and laboratory parameters (WBC count, neutrophil count, and CRP) with the bacterial count in urine and urine culture results. By identifying key predictors, we aimed to enhance early diagnosis and optimize patient management.

## 2. Materials and Methods

### 2.1. Study Design and Participants

The study was conducted at the Clinic for Infectious Diseases, University Clinical Center Tuzla (UCC Tuzla), in Tuzla, BiH. This retrospective study analyzed 200 adult patients (≥18 years) presenting upon admission with acute, community-acquired clinical symptoms indicative of a UTI. To ensure complete clarity regarding the study population, specific inclusion and exclusion criteria were strictly applied. Patients were eligible for inclusion if they were adults aged 18 years or older and presented with clinical symptoms suggestive of a urinary tract infection, such as dysuria, frequency, urgency, or flank pain. Additionally, the availability of paired samples for both automated urine flow cytometry and quantitative urine culture within the same laboratory workflow was required. Conversely, the exclusion criteria were as follows: pregnancy, pediatric status (age < 18 years), current or recent antibiotic therapy prior to or during sample collection, and the presence of long-term indwelling urinary catheters, suprapubic tubes, or nephrostomies. Furthermore, specimens that were insufficient in volume or heavily contaminated, as identified by laboratory quality control protocols, were excluded from the analysis.

Ethical approval was granted by the Institutional Ethics Committee (Approval No. 02-09/2-203-3/25).

### 2.2. Data Collection

Data was collected from 1 January 2025 to 1 January 2026, includingpatient age, gender, peripheral white blood cell (WBC) and neutrophil counts, CRP levels, urinary bacterial counts, and quantitative urine culture results.

### 2.3. Sample Collection and Laboratory Analysis

For patients presenting with symptoms suggestive of a urinary tract infection upon admission, WBC counts, neutrophil counts, serum CRP levels, routine urinalysis, and quantitative urine cultures were analyzed. Peripheral WBC and neutrophil counts were determined using the impedance method and reported in cells (10^9^/L), while serum CRP levels were quantified via an automated immunoturbidimetric assay, with spectrophotometric absorbance changes measured at 572 nm and reported in mg/L. These hematological and biochemical analyses were conducted at the Central Clinical Laboratory of the UCC Tuzla. Midstream urine specimens were collected in sterile, standardized containers. Automated urine sediment and bacterial quantification were performed at the Biochemistry Laboratory using the Sysmex UF-4000 flow cytometry analyzer (Sysmex Corporation, Kobe, Japan). Bacterial counts obtained from the automated flow cytometer were recorded in cells/µL, while traditional urine culture results were expressed in Colony-Forming Units per milliliter (CFU/mL). Throughout the study, samples were classified as positive for significant bacteriuria based on a threshold of >1200 bacteria/μL, which serves as the established internal reference value and standard operating protocol of our laboratory. This cut-off aligns with manufacturer guidelines and institutional validation workflows designed to maximize screening sensitivity and negative predictive value [11].

To further validate our institutional laboratory protocol and evaluate the precise diagnostic performance of the automated bacterial count against the traditional10^5^ CFU/mL quantitative urine culture gold standard, a Receiver Operating Characteristic (ROC) curve analysis was subsequently performed. Based on Youden’s index, the mathematically optimal post-hoc threshold calculated specifically for this cohort was ≥1367 bacteria/µL. This mathematically optimized threshold demonstrated an excellent Area Under the Curve (AUC) of 0.951, with a diagnostic sensitivity of 90.3%, a specificity of 90.7%, a positive predictive value (PPV) of 91.2%, and a negative predictive value (NPV) of 89.8%. While our primary data analysis and clinical categorization strictly relied on the established laboratory threshold of >1200 bacteria/µL, this secondary ROC analysis independently confirms the robust discriminatory power of the automated flow cytometer in our clinical setting. The analyzer was calibrated according to the manufacturer’s instructions, with two levels of quality control performed every 24 h. All procedures strictly adhered to Good Laboratory Practice (GLP) standards.

Urine cultures were processed and analyzed at the Department of Clinical Microbiology, UCC Tuzla, with results recorded after 24 and 48 h of incubation. In accordance with the institutional protocols of our microbiological laboratory, and supported by current international standards [13], a threshold of 10^5^ CFU/mL (10^8^ CFU/L)was used to define a positive urine culture.

However, we acknowledge the recent IDSA and European Federation of Clinical Chemistry and Laboratory Medicine (EFLM) European Urinalysis Guidelines [6,14], which suggest a lower threshold of 10^3^ CFU/mL (10^6^ CFU/L) as clinically significant for specific patient subgroups, including symptomatic women and patients with severe renal insufficiency. Furthermore, we note the operational distinction between screening thresholds used in automated rapid tests and confirmatory culture cut-offs. According to the EFLM guidelines [14], automated screening tools target an analytical sensitivity of at least 80% and a specificity higher than 50% to effectively rule out negative cultures, utilizing low thresholds of >10^3^ CFU/mL (>10^6^ CFU/L). When managing the diagnostic workflow, bacterial concentrations must be interpreted in conjunction with other clinical indicators, primarily leukocyturia and clinical symptoms, to determine true relevance for specific populations. While modern automated screening counters can utilize these high-sensitivity limits, our study strictly focused on confirmed, viable growth on solid media using the standard microbiological threshold of 10^5^ CFU/mL as the primary reference endpoint. This traditional cut-off remains highly specific for general epidemiological definitions in our hospital environment, though lower clinical thresholds combined with cytometric leukocyturia remain critical in targeted patient cohorts.

### 2.4. Statistical Analysis

Data normality was formally evaluated using the Shapiro–Wilk test. Continuous variables were analyzed using Spearman’s rank correlation coefficient to assess the relationship between age, laboratory parameters, and bacterial counts. Differences in the prevalence of urinary tract infections across categories of gender, bacterial count (>1200/μL or ≤1200/μL), and age were assessed using the chi-square test (*χ*^2^), with results visually represented through bar charts. To identify factors independently associated with binary outcomes (positive urine culture and bacterial count > 1200/µL), univariate and multivariate logistic regression models were employed using the ‘glm’ function. Results were expressed as Odds Ratios (OR) with 95% Confidence Intervals (CI). The diagnostic performance and discriminatory power of the automated bacterial counts were evaluated using Receiver Operating Characteristic (ROC) curve analysis, and the DeLong method was applied to compute confidence intervals for the Area Under the Curve (AUC). The post-hoc power analysis conducted in R using the pwr package showed that the regression model has a very high statistical power, with an achieved power greater than 0.98, based on the observed effect size (Cohen’s f^2^ = 0.35) and the sample size (*n* = 200). Additionally, the events-per-variable (EPV) ratio was 20.6, which was above the recommended threshold of 10 and indicated the stability of the regression estimates. Statistical significance was set at *p*< 0.05, and all analyses were performed using R (version 4.5.1, R Foundation for Statistical Computing, Vienna, Austria).

## 3. Results

**Figure 1:**
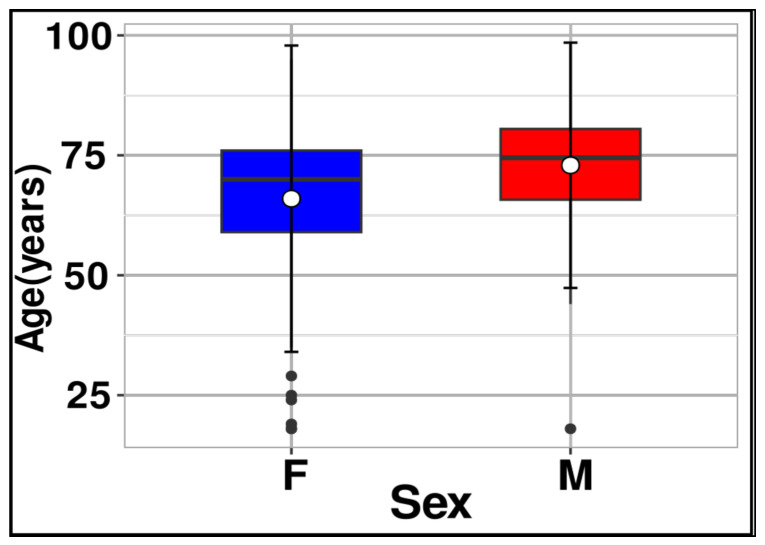
Box plot of patient age by sex (*n* = 200). The box represents the interquartile range (IQR), spanning from the first quartile (Q1, 25th percentile) to the third quartile (Q3, 75th percentile). The horizontal line within the box indicates the median age, while the white circle denotes the mean value. The vertical whiskers extend to 1.5 times the IQR, and individual black dots represent statistical outliers. Abbreviations: F, female; M, male.

The mean age of the entire study population was 68.61 ± 15.19 years (range: 18–98 years). Females were more frequently represented than males (124 vs. 76 patients, respectively). The mean age was 65.96 ± 15.97 years for females and 72.92 ± 12.78 years for males (Figure 1).

**Figure 2:**
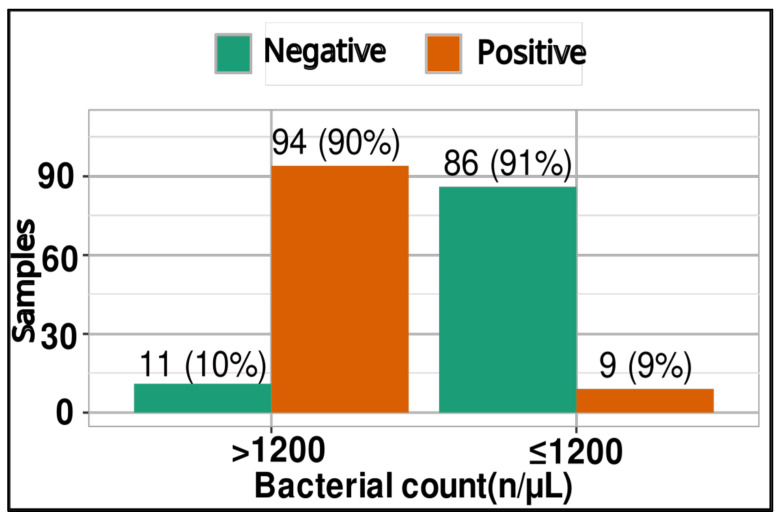
Frequency of positive urine cultures categorized by urinary bacterial count (>1200/μL vs. ≤1200/μL).

Patients with a urinary bacterial count > 1200/μL had significantly higher odds of a UTI compared to those with a bacterial count ≤ 1200/μL. Specifically, they were 83 times more likely to have a positive urine culture (OR = 83, 95% CI: 32.25–200, *p* < 0.001) (Figure 2).

**Figure 3:**
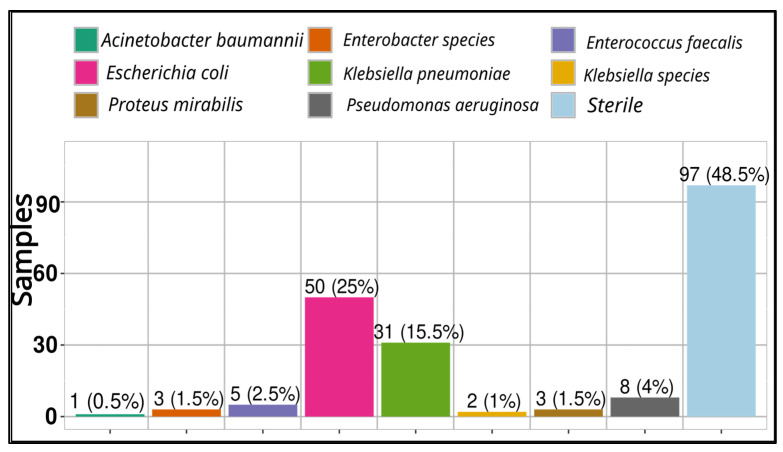
Distribution of urine culture results, including sterile samples and bacterial isolates.

Out of a total of 200 samples, 103 (51.5%) had a positive urine culture, while the remaining 97 (48.5%) were sterile (Figure 3).

**Figure 4:**
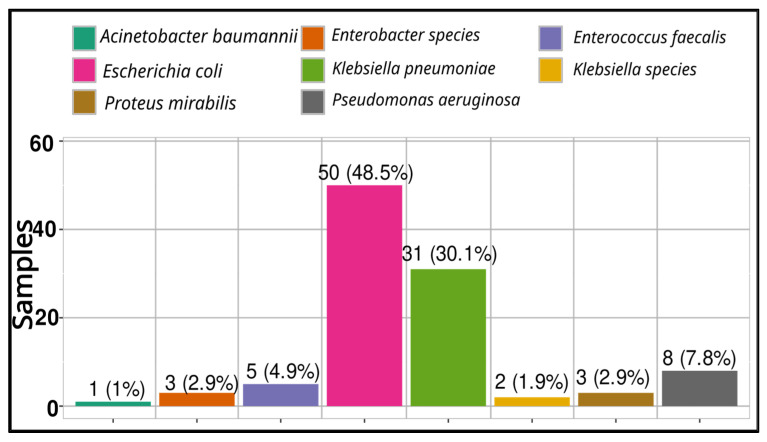
Distribution of bacterial isolates from positive urine cultures.

Figure 4 shows the frequency of isolates from urine cultures. The most frequent was *Escherichia coli* (48.5%), followed by *Klebsiella pneumoniae* (30.1%), *Pseudomonas aeruginosa* (7.8%), *Enterococcus faecalis* (4.9%), *Proteus mirabilis* and *Enterobacter species* (2.9% each), *Klebsiella species* (1.9%), and *Acinetobacter baumannii* (1%).

**Figure 5:**
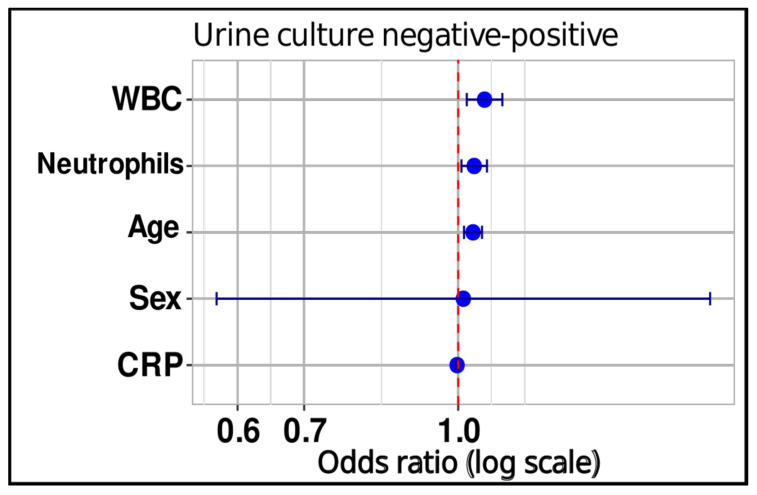
Forest plot of the logistic regression analysis demonstrating the independent predictors of a positive urine culture. The blue circular markers indicate the point estimates of the Odds Ratios (OR), while the horizontal error bars (whiskers) represent the corresponding 95% Confidence Intervals (CI). The vertical red dashed line marks the line of no effect (OR = 1.0). Parameters whose confidence intervals do not cross the reference line (WBC, Neutrophils, and Age) are statistically significant independent predictors of a positive urine culture. Conversely, the confidence intervals for CRP and Sex cross the reference line, indicating a lack of statistical significance. Abbreviations: CRP, C-reactive protein; WBC, white blood cell count.

Logistic regression analysis demonstrated that WBC count, neutrophil count, and patient age were significantly associated with the odds of UTI. Specifically, each unit increase in WBC count was associated with a 6% increase in the odds of infection (OR = 1.06, 95% CI: 1.02–1.11, *p* = 0.004), while each unit increase in neutrophils increased the odds by 4% (OR = 1.04, 95% CI: 1.01–1.07, *p* = 0.014). Patient age was also a significant factor, with each additional year increasing the odds of infection by 3% (OR = 1.03, 95% CI: 1.01–1.06, *p* = 0.001). Conversely, CRP levels and patient gender were not statistically significant predictors (CRP: OR = 0.997, 95% CI: 0.995–1.003, *p* = 0.067; gender: OR = 1.01, 95% CI: 0.572–1.79, *p* = 0.967). These findings suggest that WBC count, neutrophil count, and age are key predictors of UTI in this patient group. The results are summarized in Table 1 and illustrated in Figure 5.

**Figure 6:**
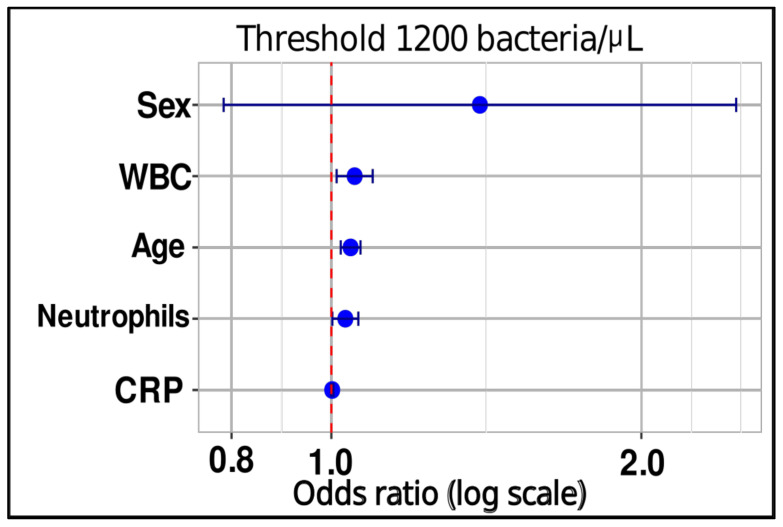
Forest plot of the logistic regression analysis showing the odds ratios (OR) on a logarithmic scale for predictors of significant bacteriuria at the laboratory threshold of 1200 bacteria/µL. The blue circular markers represent the point estimates of the odds ratios, while the horizontal error bars (whiskers) denote the corresponding 95% confidence intervals (CI). The vertical red dashed line marks the line of no effect (OR = 1.0). Predictors with confidence intervals that do not cross the reference line (WBC, Age, and Neutrophils) are statistically significant independent predictors of exceeding the 1200 bacteria/µL cytometric threshold. Abbreviations: CRP: C-reactive protein; WBC: White blood cell count.

Logistic regression analysis demonstrated that WBC count, neutrophil count, and patient age were significantly associated with the odds of a urinary bacterial count exceeding 1200/μL. Specifically, each unit increase in WBC count was associated with a 5% increase in the odds of a bacterial count > 1200/μL (OR = 1.05, 95% CI: 1.01–1.10, *p* = 0.011), while each unit increase in neutrophils increased these odds by 3% (OR = 1.03, 95% CI: 1.00–1.06, *p* = 0.035). Patient age was also a significant factor, with each additional year increasing the odds of a high bacterial count by 4% (OR = 1.04, 95% CI: 1.02–1.07, *p* < 0.001). Conversely, CRP levels and patient gender were not statistically significant predictors (CRP: OR = 1.00, 95% CI: 0.999–1.001, *p* = 0.179; gender: OR = 1.39, 95% CI: 0.786–2.47, *p* = 0.256). These findings suggest that WBC count, neutrophil count, and age are key factors associated with elevated urinary bacterial counts. The results are summarized in Table 2 and illustrated in Figure 6.

## 4. Discussion

The present study reinforces the diagnostic significance of integrating demographic factors, hematological markers and automated urine flow cytometry in the evaluation of UTIs. Our findings highlight that while certain inflammatory markers are highly predictive of infection, others, such as CRP, may have limited utility in this specific clinical context.

One of the most striking results of our study is the exceptionally high odds ratio (OR = 83) for UTI in patients with a urinary bacterial count exceeding 1200/μL via flow cytometry. This aligns with recent studies suggesting that automated flow cytometry can provide reliable results within minutes, compared to the 24–48 h required for traditional urine culture [8,15]. This is of great importance for the rapid diagnosis of UTIs, in order to avoid unnecessary antibiotic use. In our study, positive urine cultures were identified in 103 patients. *Escherichia coli* was the most frequently isolated pathogen (48.5%), which is consistent with findings from other global studies identifying it as the primary causative agent of UTI. This predominance of *E. coli* underscores its well-established role as a major uropathogen, both in community-acquired and hospital-associated infections [16,17]. However, it is noteworthy that *Klebsiella pneumoniae* was the second most prevalent isolate, accounting for 30.1% of all positive cultures. This prevalence of *K. pneumoniae* is relatively high compared to some studies [18]. Such a finding may reflect the specific patient demographic in our study, predominantly older adults (mean age 68.6 years), among whom *Klebsiella species* are more frequently associated with complicated and healthcare-associated infections. This high proportion of *K. pneumoniae* further complicates empirical treatment choices, given its well-documented potential for multidrug resistance. The present study has not included antimicrobial susceptibility testing, a major omission, especially given the expressed concern about AMR in BiH [18]. Previous studies show a disturbing pattern of multidrug resistance in *K. pneumoniae* and other Gram-negative uropathogens, including carbapenem resistance; biofilm-forming strains are also very difficult to treat [19,20]. Including resistance profiles would improve the clinical applicability and antimicrobial stewardship use of these data.

Furthermore, our analysis identified patient age as a significant independent predictor of infection. Each additional year of age increased the odds of a UTI by 3%. This is consistent with the literature, as the incidence of UTI is known to increase substantially with age in both men and women. These geriatric UTIs are frequently complicated, often involving immunosenescence alongside structural or functional abnormalities of the genitourinary tract, which predispose this demographic to frequent infections [21,22]. Interestingly, gender did not emerge as a significant predictor in our multivariate model (*p* = 0.967). Although females traditionally exhibit a higher overall incidence of UTIs in general populations, this lack of statistical difference in our predictive models (Figure 5 and Figure 6) may reflect the specific elderly-dominated profile of our study population, where age-related risks potentially outweigh traditional gender-based differences. Furthermore, this outcome is likely influenced by the clinical setting of our cohort, which comprised severely symptomatic patients admitted to a tertiary care infectious disease clinic. In such specialized settings, clinical severity and bacterial density tend to equilibrate between sexes, with objective markers like advanced age and peripheral leukocytosis emerging as the dominant independent drivers of infection risk rather than biological sex alone. In community-dwelling older adults, the incidence and prevalence of UTI vary with age. For instance, the baseline incidence of UTI has been reported at approximately 0.07 per person-year, while the prevalence in one cohort study among women older than 65 years was found to be approximately 16.5% over a 6-month period [23]. Griebling reported that the annual incidence of UTI in men ranges from 0.05 among those aged 65 to 74 years, increasing to approximately 0.08 in men aged 85 and older [24]. One biological reason for the higher incidence of UTIs in elderly women is the change in vaginal flora resulting from declining estrogen levels, which predisposes postmenopausal women to colonization by uropathogens, although oral estrogen replacement therapy has not been consistently associated with the prevention of UTIs in this population [25]. Conversely, in aging men, prostatic hypertrophy causing urinary retention and high postvoid residuals is strongly associated with the development of UTIs—a clinical condition that becomes increasingly prevalent with advancing age [21]. This suggests that while gender-specific factors like estrogen depletion and prostatic changes play a role, the overall systemic aging process, including structural urinary stasis, remains a dominant driver of infection risk across both genders in our study group.

Interestingly, while peripheral WBC and neutrophil counts were significantly associated with both positive cultures and high bacterial loads, CRP levels did not reach statistical significance (*p* = 0.067). This suggests that in our study population, specific cellular responses in the blood are more sensitive indicators of a UTI than the general inflammatory marker CRP. The lack of statistical significance for CRP in our cohort may be attributed to the fact that it is a non-specific marker of systemic inflammation, which may not be sufficiently elevated in localized UTIs and can be influenced by other underlying comorbidities in our predominantly older study population (mean age 68.61 years). This is consistent with the literature; in the study by Shi et al., statistical analysis showed that CRP exhibited significant predictive potential for upper urinary tract bacteria, whereas it was a poor predictor for lower urinary tract infections [26]. Similarly, Stalenhoef et al., who investigated the duration of antibiotic treatment in patients with urinary tract infections, demonstrated that CRP was an unreliable parameter for monitoring these patients [27]. To overcome these diagnostic limitations of CRP, serum procalcitonin (PCT) has increasingly emerged as a superior alternative biomarker for anatomical localization. Accumulating evidence demonstrates that PCT levels scale directly with the severity of renal parenchymal inflammation, yielding significantly higher clinical specificity than CRP when differentiating acute pyelonephritis from localized lower tract infections like cystitis [28]. Consequently, incorporating PCT alongside automated cytometric profiles could substantially reduce diagnostic ambiguity in tertiary emergency settings.

In contrast, peripheral WBC and neutrophil counts may reflect an earlier or more specific cellular immune response to the uropathogens present in our patient cohort.

Neutrophils, the primary white blood cells in human peripheral blood, are characterized by high production rates in the bone marrow and a short lifespan of about 36 h. While they normally exit the circulation via apoptosis, their survival is significantly enhanced in response to infectious stimuli. This extension is driven by exposure to microbial products such as lipopolysaccharides (LPS), as well as various complement proteins and inflammatory cytokines [29]. Although neutrophil chemotaxis diminishes with advancing age, aging is paradoxically linked to heightened tissue recruitment and activation. Such dysfunctional activation can extend the presence of neutrophils within tissues, intensifying both local and systemic inflammation in the elderly [30]. This finding is particularly relevant as it challenges the routine reliance on CRP for UTI screening, suggesting instead that clinicians should prioritize WBC and neutrophil differentials when evaluating suspected cases.

Despite its significant findings, several limitations of this study should be noted. First, the retrospective design inherently limits our ability to control for all potential confounding factors or to establish a direct causal relationship. Second, the study was conducted at a single clinical center, which may affect the generalizability of the results to other healthcare settings with different patient demographics or laboratory protocols. Third, the sample size of 200 patients, while sufficient for our primary statistical analyses, may have lacked the power to identify smaller effects, such as the borderline significance observed for CRP levels. Regarding the borderline significance of CRP (*p* = 0.067), the post-hoc power evaluation indicates that this near-significant trend is likely a reflection of the inherent biological variability of inflammatory kinetics in severe UTIs, rather than an underpowered sample size. These statistical validation metrics reinforce the reliability of our findings despite the retrospective nature of the study design.

Furthermore, the high mean age of the female cohort in this study (65.96 ± 15.97 years) indicates that the vast majority of these patients were postmenopausal. Estrogen deficiency is a key physiological factor that increases susceptibility to UTIs, mediated by urogenital mucosal atrophy and alterations in the protective vaginal microbiota. Due to the retrospective, laboratory-focused design of this study, specific details regarding menopausal status or estrogen replacement therapy were unavailable, which represents a limitation. Nonetheless, the age profile strongly underlines the clinical importance of optimizing rapid screening tools like flow cytometry in this high-risk, elderly demographic. Additionally, we did not categorize patients into uncomplicated versus complicated UTIs, which could have provided deeper insights into the predictive value of inflammatory markers across different clinical phenotypes. A key characteristic of the present study is that data collection was restricted to a tertiary care referral center. In accordance with local clinical pathways, uncomplicated, localized lower tract infections (such as community-acquired cystitis) are managed at the primary care level and rarely undergo tertiary microbiological screening. Therefore, our cohort naturally consists of patients presenting with severe systemic manifestations or complicated courses (e.g., suspected community-acquired pyelonephritis or urosepsis), which represent the higher severity spectrum of the EAU classification. This institutional selection bias explains the high baseline inflammatory markers observed in our regression models (Table 1 and Table 2). While the lack of a primary care comparative cohort limits the generalizability of our findings to simple cystitis, it highlights the clinical utility of our automated bacterial (>1200/µL) thresholds within a specific, high-risk tertiary population. In these complex phenotypes, automated screening serves as a robust tool to rapidly rule out negative cultures, thereby optimizing the diagnostic workflow in emergency and specialized inpatient departments.

While traditional urinalysis parameters such as nitrites, hematuria, and urine pH provide valuable clinical screening information, they were excluded from the final multivariate predictive model. This decision was made to prevent statistical multicollinearity with the absolute cytometric bacterial counts and to account for the compromised diagnostic sensitivity of chemical test strips (e.g., nitrite conversion limitations) frequently observed in acutely ill, hospitalized cohorts. To properly contextualize the diagnostic utility of automated flow cytometry, its inherent false-positive and false-negative rates must be addressed. Analytical false-positive results are frequently driven by non-bacterial particulate matter, including crystal artifacts, amorphous sediments, spermatozoa, and cellular debris, which can closely mimic bacterial scattering signals on the analyzer [12].

Furthermore, pre-analytical confounding factors significantly impact specific vulnerable populations. Elderly patients, particularly those with indwelling urinary catheters or suffering from urinary incontinence, present a substantially higher risk of sample contamination due to perineal colonization or biofilm fragmentation. In these cohorts, automated screening often overestimates bacterial concentrations (>1200/μL), resulting in lower clinical specificity. Nevertheless, the diagnostic performance metrics observed in our cohort align closely with widely published validation studies, which typically report screening sensitivities between 85% and 95% [31]. This challenge underscores the ongoing debate raised by Biguenet et al. (2025) regarding the urgent need to reevaluate and optimize gender- and age-specific cytometric thresholds within geriatric medicine to prevent the overdiagnosis of active infection in the presence of asymptomatic bacteriuria [15].

In comparison with published validation literature, which reports a wide range of screening sensitivities (80–96%) and specificities (60–88%), our cohort’s ROC-validated metrics (sensitivity 90.3%, specificity 90.7%) align closely with the upper tier of international performance standards. This robust performance confirms that while flow cytometry cannot replace culture identification, it serves as an exceptional rule-out screening tool when integrated with host clinical indicators [8]. The automated urine flow cytometer delivers precise quantitative results in under one minute per specimen. This rapid turnaround time significantly enhances screening efficiency by quickly ruling out negative samples, although traditional quantitative cultures remain mandatory for positive specimens to achieve definitive pathogen identification and antibiotic susceptibility testing.

Future prospective, multicenter studies with larger cohorts are needed to validate these findings and to establish more precise diagnostic algorithms that integrate flow cytometry with systemic markers [8].

Integrating automated urinary flow cytometry (FCM) into resource-limited healthcare settings, such as BiH, presents a compelling cost-effectiveness rationale despite high initial capital equipment costs. Standard quantitative urine culture workflows remain resource-intensive, with estimated micro-costing ranges between €4.00 and €6.00 per sample when factoring in selective agar plates, confirmatory reagents, manual labor, and incubator energy consumption. In contrast, the consumable cost of an automated FCM screening run is substantially lower, typically ranging from €0.50 to €1.00 per specimen. In our study cohort, 48.5% of all processed urine samples were confirmed to be sterile. By utilizing FCM as a rapid, high-sensitivity ‘rule-out’ tool, nearly half of the total sample volume could be safely excluded from subsequent microbiological culture within minutes of arrival. This diagnostic stewardship strategy translates into massive direct cost savings regarding laboratory consumables and significantly reduces the manual workload of specialized laboratory personnel. For less developed healthcare networks or regional centers facing infrastructural and staffing constraints, implementing centralized automated counters provides a scalable solution to optimize constrained budgets.

These findings strongly support the principles of diagnostic stewardship, suggesting that integrating rapid flow cytometry with specific hematological parameters and patient age can provide clinicians with objective data to make more informed decisions. Furthermore, reducing the diagnostic turnaround time from 48 h to less than an hour curtails inappropriate empirical antimicrobial prescriptions, yielding indirect hospital savings by reducing antibiotic-associated adverse events, optimizing constrained budgets, and mitigating the long-term economic burden of antimicrobial resistance in developing regions.

Looking ahead, innovations in molecular diagnostics and automated analytical tools offer promising avenues to further refine this clinical workflow. Additional innovations, such as multiplexing technology for molecular diagnostics (e.g., PCR), could allow clinicians to identify pathogens and resistance patterns on the same day of testing, allowing for more timely and targeted therapeutic interventions in resource-scarce settings [32,33]. Furthermore, new biomarker methods for distinguishing between upper and lower urinary tract infections, including Neutrophil Gelatinase-Associated Lipocalin (NGAL) and procalcitonin, warrant investigation in clinical decision algorithms [34]. Ultimately, future research should leverage artificial intelligence (AI) coupled with flow cytometry methods to continuously enhance diagnostic accuracy and screening efficiency [35].

## 5. Conclusions

Our study demonstrates that automated urine flow cytometry, specifically the measurement of urinary bacterial count, serves as a powerful predictor of UTI. We identified a urinary bacterial count threshold of >1200/μL as a critical indicator, with patients exceeding this level being 83 times more likely to have a positive urine culture. Furthermore, logistic regression analysis revealed that WBC count, neutrophil count, and patient age are significant independent predictors of both UTI occurrence and high bacterial loads. Conversely, CRP levels and gender did not show a statistically significant predictive value in this cohort.

These findings suggest that incorporating rapid screening tools like flow cytometry, alongside clinical and demographic factors such as age and peripheral blood counts, can significantly enhance early diagnostic accuracy. In clinical settings like BiH, where empirical antibiotic therapy is common, the application of these predictors could optimize patient management, reduce the reliance on broad-spectrum antibiotics while awaiting culture results, and ultimately help mitigate the growing challenge of antimicrobial resistance.

## Figures and Tables

**Table 1:** Logistic regression results for parameters associated with a positive urine culture.

Parameter	OR	95% CI	*p*-Value
WBC count	1.063	1.020–1.111	**0.004**
CRP	0.997	0.995–1.003	0.067
Neutrophil count	1.042	1.011–1.070	**0.014**
Age	1.030	1.010–1.064	**0.001**
Sex	1.015	0.572–1.797	0.967

Note: OR, Odds Ratio; CI, Confidence Interval; VIF, Variance Inflation Factor. Bold *p*-values indicate statistical significance (*p* < 0.05). For continuous variables (WBC count, CRP, Neutrophil count, and Age), the OR represents the change in odds per single-unit increase in each parameter, explaining why values are close to 1.0 despite high statistical significance. For the categorical variable (Sex), the male group was compared against the female reference group. Multicollinearity diagnostics revealed that all VIF values were close to 1 (ranging from 1.04 to 1.20). Specifically, the VIF values for white blood cell and neutrophil counts confirmed the absence of any significant multicollinearity, validating the inclusion of both parameters within the final regression model.

**Table 2:** Logistic regression results for parameters associated with a urinary bacterial count > 1200/μL.

Parameter	OR	95% CI	*p*-Value
WBC count	1.050	1.010–1.100	**0.011**
CRP	1.000	0.999–1.001	0.179
Neutrophil count	1.030	1.000–1.060	**0.035**
Age	1.040	1.020–1.070	**0.001**
Sex	1.390	0.786–2.470	0.256

Note: OR, Odds Ratio; CI, Confidence Interval. Bold *p*-values indicate statistical significance (*p* < 0.05), showing a significant effect of the parameter on the likelihood of the urinary bacterial count exceeding 1200/µL. For continuous variables (WBC count, CRP, Neutrophil count, and Age), the OR represents the change in odds per single-unit increase in each parameter. For the categorical variable (Sex), the male group was compared against the female reference group.

## Data Availability

The de-identified datasets generated during and/or analyzed during the current study are available from the corresponding author on reasonable request.

## Abbreviations

The following abbreviations are used in this manuscript:

AI	artificial intelligence
AMR	antimicrobial resistance
AUC	area under the curve
BiH	Bosnia and Herzegovina
CDC	Centers for Disease Control and Prevention
CFU/mL	colony-forming units per milliliter
CFU/L	colony-forming units per liter
CI	confidence interval
CRP	C-reactive protein
cUTI	complicated urinary tract infection
EAU	European Association of Urology
EFLM	European Federation of Clinical Chemistry and Laboratory Medicine
ESCMID	European Society of Clinical Microbiology and Infectious Diseases
FCM	flow cytometry
FL	fluorescent light
FSC	forward scatter
GLP	Good Laboratory Practice
IDSA	Infectious Diseases Society of America
IQR	interquartile range
LPS	lipopolysaccharides
NGAL	neutrophil gelatinase-associated lipocalin
NPV	negative predictive value
OR	odds ratio
PCR	polymerase chain reaction
PPV	positive predictive value
PCT	procaltinonin
ROC	receiver operating characteristic
SSC	side scatter
UCC Tuzla	University Clinical Center Tuzla
uUTI	uncomplicated urinary tract infection
UTI	urinary tract infection
VIF	variance inflation factor
WBC	white blood cell
